# *In Vitro* and *In Vivo* Toxicity Studies on* Cymbopogon giganteus *Chiov. Leaves Essential Oil from Benin

**DOI:** 10.1155/2020/8261058

**Published:** 2020-01-28

**Authors:** Habib Toukourou, Francine Uwambayinema, Yousof Yakoub, Birgit Mertens, Anatole Laleye, Dominique Lison, Joelle Quetin-Leclercq, Fernand Gbaguidi

**Affiliations:** ^1^Laboratoire de Chimie Pharmaceutique Organique, Ecole de Pharmacie, Faculté des Sciences de la Santé, Université d'Abomey-Calavi, Campus du champ de Foire, 01BP 188 Cotonou, Benin; ^2^Pharmacognosy Research Group, Louvain Drug Research Institute, Université Catholique de Louvain, B1 7203 Av. E. Mounier 72, B-1200 Brussels, Belgium; ^3^Louvain Centre for Toxicology and Applied Pharmacology, Institut de Recherche Expérimentale et Clinique, Université catholique de Louvain, B1.57.06 Av. Hippocrate 57, B-1200 Brussels, Belgium; ^4^Scientific Direction of Chemical and Physical Health Risks, Sciensano, Juliette Wytsmanstraat 14, 1050 Brussels, Belgium; ^5^Unité de Biologie Humaine, Laboratoire de Cytogénétique et de Biologie Moléculaire, Faculté des Sciences de Santé, Université d'Abomey-Calavi, Campus du champ de Foire, 01BP 188 Cotonou, Benin

## Abstract

*Cymbopogon giganteus* Chiov. (Poaceae) is a medicinal plant used to treat various diseases in traditional medicine in several African countries. The present study aims to evaluate the oral and inhalation toxicity as well as the mutagenic effects of the essential oil of *Cymbopogon giganteus *leaves (EOCG) from a sample collected in Benin. Mutagenic potential was assessed by the Ames test using *Salmonella typhimurium *strains TA98 and TA100. Oral acute toxicity was carried out by administration of a single dose of 2000 mg/kg b.w. to Wistar rats while oral subacute toxicity was assessed by daily administration of 50 and 500 mg/kg of EOCG for 28 days. Finally, inhalation toxicity was assessed by administration of a single dose of 0.125%, 0.5%, 2% or 5% v/v of EOCG emulsions in 0.05% v/v lecithin solution in sterile water for the first experiment, and in a second one by administration of single dose of 0.125% or 0.5% v/v. A broncho-alveolar lavage was performed after 3 h or 24 h, respectively. The results show that EOCG is not mutagenic on* Salmonella typhimurium *strains at the highest concentration tested (200 *μ*g/plate). In the acute oral toxicity study, EOCG induce neither mortality nor toxicity, showing that the LD_50 _is greater than 2000 mg/kg. The subacute oral toxicity study at both doses did not show any significant difference in body weight, relative organ weight, hematological and/or biochemical parameters or histopathology as compared to the control group. EOCG induced mortality and inflammation in lungs 3 h after administration of a single dose of 5% or 2% v/v. Single doses of 0.125% or 0.5% v/v did not induce inflammation, cell recruitment nor cytotoxicity in lungs 3 h or 24 h after administration, suggesting safety at these concentrations. This first report on the *in vivo* toxicity will be useful to guide safe uses of EOCG.

## 1. Introduction

Medicinal herbs and their related products are traditionally used by at least 80% of the population in developing countries to solve or improve their health problems [1]. Their uses are based on ancestral habits transmitted from generation to generation. Unlike modern medicines containing one or a few well-known active substances, these medicines contain a multitude of bioactive substances in very variable proportions, which depend on several factors [2]. This explains the difficulties of their standardization in terms of efficacy. The toxicity of these preparations, especially upon long-term application, is often neglected [3, 4]. In this context, toxicological evaluation appears obviously a crucial step in determining safety and to support the traditional use of these medicines.

*Cymbopogon giganteus *Chiov. (Poaceae) also call “Citronelle de Madagascar” is a grass, which can grow up to 2-3 m, spreading in regions of tropical Africa. This plant is used in traditional medicine against various diseases such as skin disorders [5], mental illness, broncho-pulmonary affections, bilharzia, jaundice, cold, conjunctivitis, migraine, dermatoses, rheumatic pains, childhood coughs and hepatitis [6, 7]. The essential oil of *Cymbopogon giganteus* (EOCG) got by hydro distillation of its leaves showed good antibacterial and anti-inflammatory properties and is sold for these activities [8–11]. We analyzed the composition of a sample of EOCG originating from Benin. It was characterized by the presence of limonene, carvone, carveol and *p*-menthane derivatives as major compounds [12], like other samples from other origins [11, 13, 14]. Using essential oils and particularly by the oral route can, however, produce serious adverse effects [15] which can be predicted by performing toxicological tests.

To the best of our knowledge, there is no data in the literature on *in vivo *oral and pulmonary toxicity of EOCG, nor on its mutagenicity, which may be required to predict its safety and/or toxic effects following exposure.

Our goal being the use of essential oil of *Cymbopogon giganteus *(EOCG) as antiseptic and antibacterial against sore throat caused by bacteria and viruses, we focused our studies on *in vitro* mutagenicity (by Ames test), acute and subacute oral toxicity and acute pulmonary toxicity in Wistars rats.

## 2. Materials and Methods

### 2.1. Animals and Plant Material

For oral toxicity testing, Wistar albino rats aged of 10–12 weeks were obtained from the house facilities of UAC-FSS (Université d'Abomey Calavi, Faculté des Sciences de la Santé, Cotonou, Bénin) while for pulmonary toxicity, eight-week-old female Wistar rats were purchased from Janvier Labs (St Berterin, France). All animals were acclimatized for one week, kept with sterile rodent feed with free access to water and housed in positive pressure air-conditioned units (25°C, 50% relative humidity) on 12 h light/dark cycle.

Fresh leaves of *Cymbopogon giganteus* Chiov. were collected in Parakou areas (9°20′N, 2°37′E) in November 2016. Crops were identified by the Herbier National du Bénin (Université Abomey Calavi) where a voucher specimen was deposited under number AA6680/HNB. EOCG was obtained by hydro-distillation using a Clevenger apparatus of air-dried leaves and stored at 4°C.

### 2.2. Bacterial Reverse Gene Mutation Test (Ames Test)

The *in vitro* mutagenic potential of EOCG were evaluated in the bacterial reverse gene mutation test as stated in guideline 471 of the organization for economic Co-operation and Development (OECD) [16], with slight modifications as described by [17]. Although normally 5 different bacterial strains should be used, the test was performed in two strains only, i.e. *Salmonella typhimurium *TA98 and TA100, as this combination already allows to identify up to 90% of mutagens [18]. After cultivation of the *Salmonella *bacteria (Moltox, Boone, USA) overnight, 100 *µ*l of the bacterial suspension was mixed with 100 *µ*l of the EOCG emulsion, 500 *µ*l sodium phosphate buffer pH 7.4 and 2 mL overlay agar enriched with a histidine-biotine solution. Emulsions of EOCG were prepared at 0.2% v/v (0.2% of EOCG and 0.02% v/v of tween 80 in sterile water), resulting in a highest concentration tested of 200 *μ*g/plate. In total, five concentrations were tested (200, 50, 20, 5, and 2 *μ*g/plate). To evaluate the impact of metabolic activation, the buffer was replaced by a 5% S9 metabolization mix (prepared from lyophilized rat liver S9 mixed with nicotinamide adenine dinucleotide phosphate (NADPH) regenerating system—both from Moltox). The resulting mixture was poured onto a minimal glucose agar plate (E&O Laboratories Ltd., Bonnybridge, United Kingdom) and incubated for 48 h at 37°C (Binder, Tuttlingen, Germany). Triplicate plates were poured for each test condition. Positive, negative and solvent control plates were prepared in parallel with the test substance plates. A solution of Tween 80 (20 *μ*g/plate) was used as solvent control. For the tests without S9 metabolizing mix, sodium azide (2 *µ*g/plate) and 4-nitroquinoline-*n*-oxide (0.2 *µ*g/plate) were used as a positive control for strains TA100 and TA98, respectively. 2-Aminoanthracene (1 *µ*g/plate) was used as positive control for the tests with metabolic activation. All positive control substances and tween 80 were purchased from Sigma-Aldrich (Saint-Louis, USA). After incubation, the plates were inspected for cytotoxicity and precipitation by examining the background lawn using a light microscope (Zeiss, Oberkochen, Germany) at 40x total magnification. All plates were subsequently scored using a manual colony counter (Sigma-Aldrich, Saint-Louis, USA). Individual plate counts were recorded as well as the mean number of revertant colonies per plate. Thereafter, the standard deviation (SD) was calculated. A substance is considered being mutagenic when the number of colonies obtained for the test substance/number of colonies obtained for the solvent control is greater than 2 (*N* > 2) and a dose-dependent effect is observed.

### 2.3. Toxicity Assays

#### 2.3.1. Study Compliance

Acute and subacute toxicity studies were performed according to OECD Principles and the internationally accepted principles for laboratory animal use and care (NIH publication No. 85-23, revised 2010). Acute pulmonary toxicity test was performed under the ethical standards at Université catholique de Louvain, Comité d'Ethique pour l'Expérimentation Animale, Secteur des Sciences de la Santé, Brussels, Belgium (No. LA1230312).

#### 2.3.2. In Vivo Acute Oral Toxicity

The acute toxicity test was carried out based on the OECD guidelines for chemicals testing, sections 4-423-(limit test), adopted in 2008 [19]. Six female Wistar rats weighing 163 ± 7.2 g were divided into two groups of 3 rats each. The experimental group was gavaged with a single dose of 2000 mg/kg b.w. of essential oil diluted in 2 mL maize oil while the control group was treated with 2 mL of maize oil only. The animals were observed for eight hours just after the administration and once daily up to 14 days. The monitoring was based on general behavioral changes, body weight evolution, mortality and any other toxicity signs. At the end of the experience (day 15), 0.1 mL of thiopental (100 mg/mL) was injected intraperitoneally to euthanize the animals to collect blood samples in tubes for biochemical examinations.

#### 2.3.3. In Vivo Subacute Oral Toxicity Test

The subacute toxicity test was carried out as described in OECD 407 guidelines [20]. 30 Wistars rats (15 males and 15 females) were used and distributed in three groups (5 males and 5 females/group): a control (group 1) which received 2 mL of maize oil (vehicle) daily and two dose levels groups (group 2 and group 3) of EOCG (50 and 500 mg/kg body weight/day) diluted in 2 mL maize oil. EOCG was administrated daily by gavage for 28 days. During the experiment, the animals were observed for signs of toxicity and mortality twice a day. Observations were focused on changes in the skin and fur, eyes, mucus, respiratory tract, autonomic and somato-motor activity and behavioral patterns. Body weight was recorded every five days and blood samples were collected at day 15. At the end (day 29), rats were starved overnight (12 hours) but with free access water. They were then anesthetized, sacrificed by an overdose of thiopental and blood samples were collected in tubes without anticoagulant for biochemical parameters analysis and in EDTA pre-coated tubes to obtain the whole blood for hematological determinations. Different organs (stomach, liver and kidneys) were carefully excised and their absolute weights were determined. The relative organ weight was then calculated as follows: (absolute organ weight (g)/body weight of rat on the sacrifice day (g)) × 100. Livers and kidneys were washed with regular saline and were placed in 3.65% paraformaldehyde (Sigma-Aldrich, St Louis, Missouri, USA) in phosphate buffered saline (PBS) for later histological analysis by staining with hematoxylin-eosin.

#### 2.3.4. Acute Pulmonary Toxicity Test

Pulmonary toxicity was assessed after oropharyngeal aspiration of EOCG. This method is well known to assess the pulmonary toxicity of compounds or particles as those induced by asbestos, crystalline silica or bleomycin [21–24]. Briefly, 300 *μ*l of emulsion in 0.05% lecithin solution in sterile water of EOCG at different concentrations were administrated directly by oro-pharyngeal aspiration. All experiments were performed with groups of 5 rats: a control group (vehicle) and groups which received different concentrations. In the first set of tests, 0.125%, 0.5%, 2%, and 5% v/v of emulsions of EOCG were administrated and rats were sacrificed 3 h later. In a second set of tests, single doses of 0.125% and 0.5% v/v emulsion of EOCG were administrated with sacrifice of rats 24 h after. The control group received 300 *μ*l of vehicle (0.05% v/v of lecithin solution (Lipoid S100 (LIPOID GMBH) in sterile water) for each experiment. All rats were euthanized by intraperitoneal injection of 12 mg sodium pentobarbital (Certa, Braine-l'Alleud, Belgium). Broncho-alveolar lavage fluid (BALF) was obtained by inserting a cannula in trachea and infusing the lungs with 10 mL of NaCl 0.9%. This fluid was submitted to centrifugation for 10 min at 4°C (240 g). Cell-free supernatant was used for determination of total proteins and lactate dehydrogenase (LDH) activity [25]. The cells pellet was re-suspended in PBS and was counted after staining with Turch (crystal violet 1%, acetic acid 3%). For the second set of tests, cell differentiation was measured for 200 cells counted by light microscopy after cytocentrifugation (CYTOSPIN3, SHANDON) and Diff-Quick staining (Polysciences, Warrington, UK).

### 2.4. Biochemical Parameters

Serum concentrations of urea, creatinine, alanine aminotransferase (ALAT), alkaline phosphatase (PAL) and aspartate aminotransferase (ASAT) were determined using an automatic analyzer (BS-200 Minidray) with specific kits (Cypress Diagnostics, Langdorp, Belgium). For hematological analysis, hematocrit (HCT), hemoglobin (Hb), erythrocytes, leukocytes, and platelets (PLT) counts were performed on a XS-500i Sysmex automated hematology analyzer.

### 2.5. Statistics

All results are expressed as mean ± standard errors on the mean (SEM) and statistical analyses were performed with GraphPad Prism 5.0 (GraphPad Software Inc., San Diego, CA, USA) and/or Microsoft excel 2016. For acute toxicity, *t*-test was used to compare data. For subacute toxicity test, data from male and female animals were analyzed separately. Differences between control and treated groups were evaluated using one-way analysis of variance (ANOVA) followed by a Dunnett's multiple comparison for subacute and pulmonary toxicity. Statistical significance was considered at *p* < 0.05.

## 3. Results

### 3.1. Mutagenicity Assay

The results of the mutagenicity assay are depicted in Table 1, which shows that EOCG was not mutagenic nor in TA 98 nor in TA100 strains neither in presence or absence of S9 metabolic activation system.

### 3.2. Acute Toxicity Test

**Figure 1:**
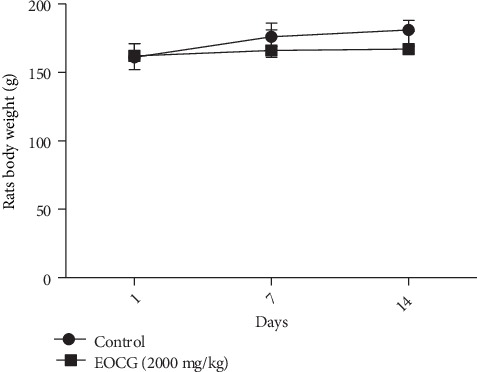
Rat body weight during the acute toxicity experimentation with EOCG at 2000 mg/kg; *n* = 3.

**Figure 2:**
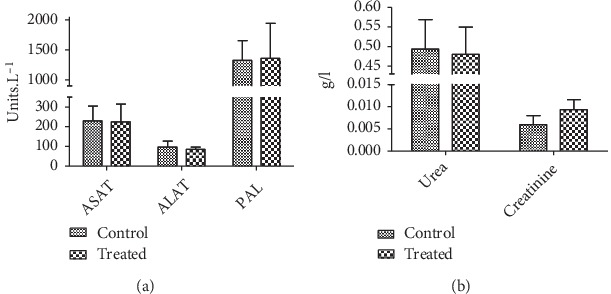
Biochemical parameters at the end of acute toxicity test with EOCG (2000 mg/kg). Panel (a) Liver biochemicals parameters; (b) kidney biochemicals parameters; ASAT (aspartate aminotransferase); ALAT (alanine aminotransferase); PAL (alkaline phosphatase). Data are expressed as mean ± SEM; *n* = 3.

The oral toxicity was assessed using the “limit acute toxicity test” based on the OECD guidelines. The 2000 mg/kg dose tested did not cause death nor any abnormality in the general behavior, except symptoms such as torpor just after gavage. As shown in Figure 1, there was no significant difference between the body weights of the two groups of rats after 14 days. Furthermore, as shown in Figure 2, the biochemical parameters did not show any significant change in the groups of treated animals compared with the controls.

### 3.3. Sub Chronic Toxicity Test

**Figure 3:**
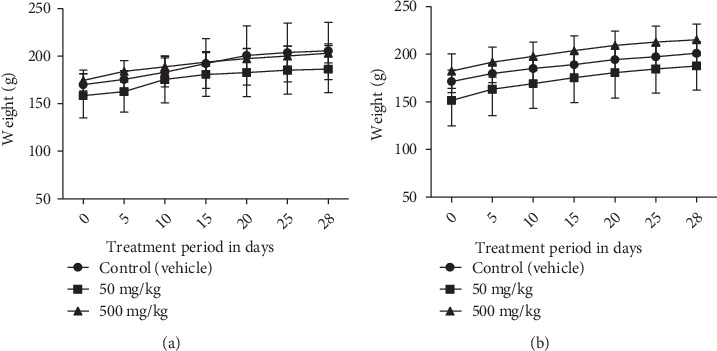
Changes of rat body weight during the subacute toxicity experimentation with EOCG at 50 and 500 mg/kg; (a) females, *n* = 5; (b) males, *n* = 5.

**Figure 4:**
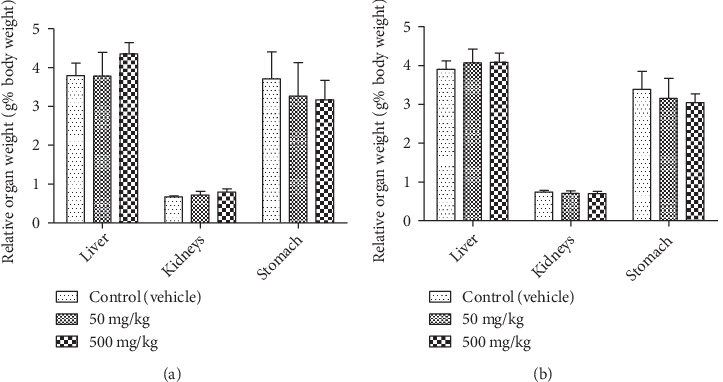
Relative organ weights of rat organs at the end of treatment with EOCG (oral doses of 50 and 500 mg/kg body weight for 28 days). (a) Females, *n* = 5; (b) males, *n* = 5. Data are expressed as mean + SEM.

**Figure 5:**
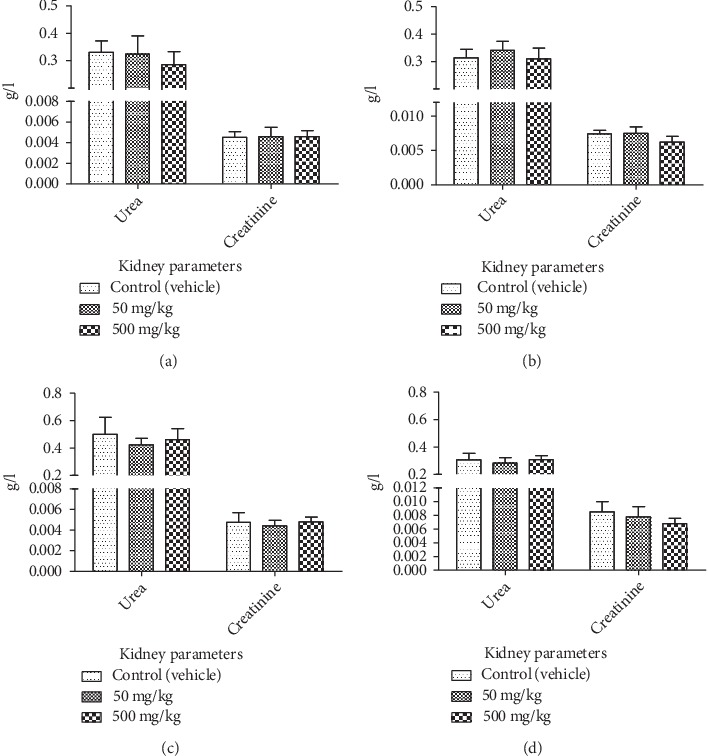
Effect of chronic oral administration of EOCG on biochemical kidney parameters in rats. The EOCG was given daily by oral route to groups of Wistar rats at the following doses: 0 (Control), 50 and 500 mg/kg b.w. for 28 days. Biochemical parameters were measured at day 15 and after 28 days of treatment. (a) Urea and creatinine at day 15 of treatment for female rats; (b) urea and creatinine at the end of treatment for female rats; (c) urea and creatinine at day 15 of treatment for male rats; (d) urea and creatinine at the end of treatment for male rats. Data are expressed as mean ± S.E.M for *n* = 5.

**Figure 6:**
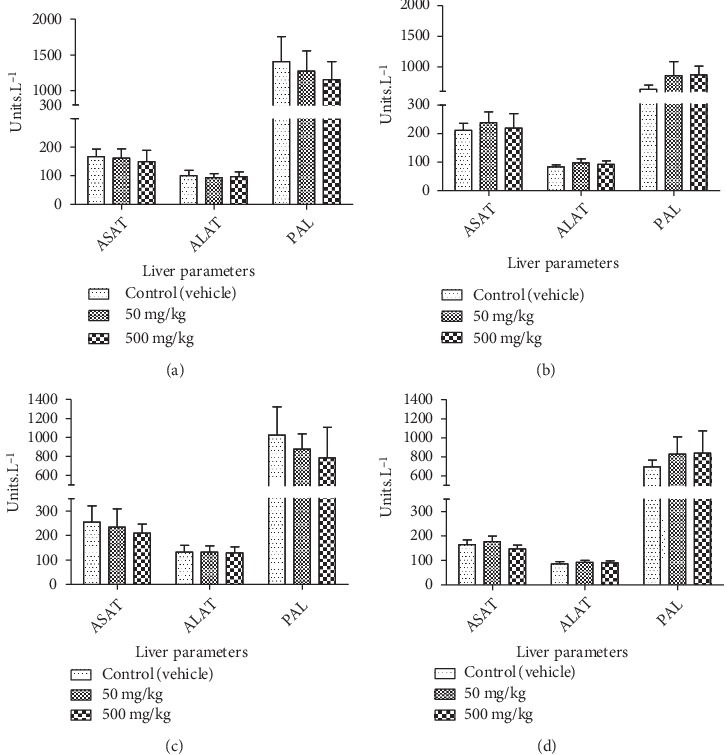
Effect of chronic oral administration of EOCG on biochemical liver parameters in rats. The EOCG was given daily by oral route to groups of Wistar rats at the following doses: 0 (Control), 50 and 500 mg/kg for 28 days. Biochemical parameters were measured at day 15 and after 28 days of treatment. (a) ASAT (aspartate aminotransferase); ALAT (alanine aminotransferase); PAL (alkaline phosphatase) at day 15 of treatment for female rats; (b) ASAT (aspartate aminotransferase); ALAT (alanine aminotransferase); PAL (alkaline phosphatase) at the end of treatment for female rats; (c) ASAT (aspartate aminotransferase); ALAT (alanine aminotransferase); PAL (alkaline phosphatase) at 15 days of treatment for male rats; (d) ASAT (aspartate aminotransferase); ALAT (alanine aminotransferase); PAL (alkaline phosphatase) at the end of treatment for male rats. Data are expressed as mean ± S.E.M for *n* = 5.

**Figure 7:**
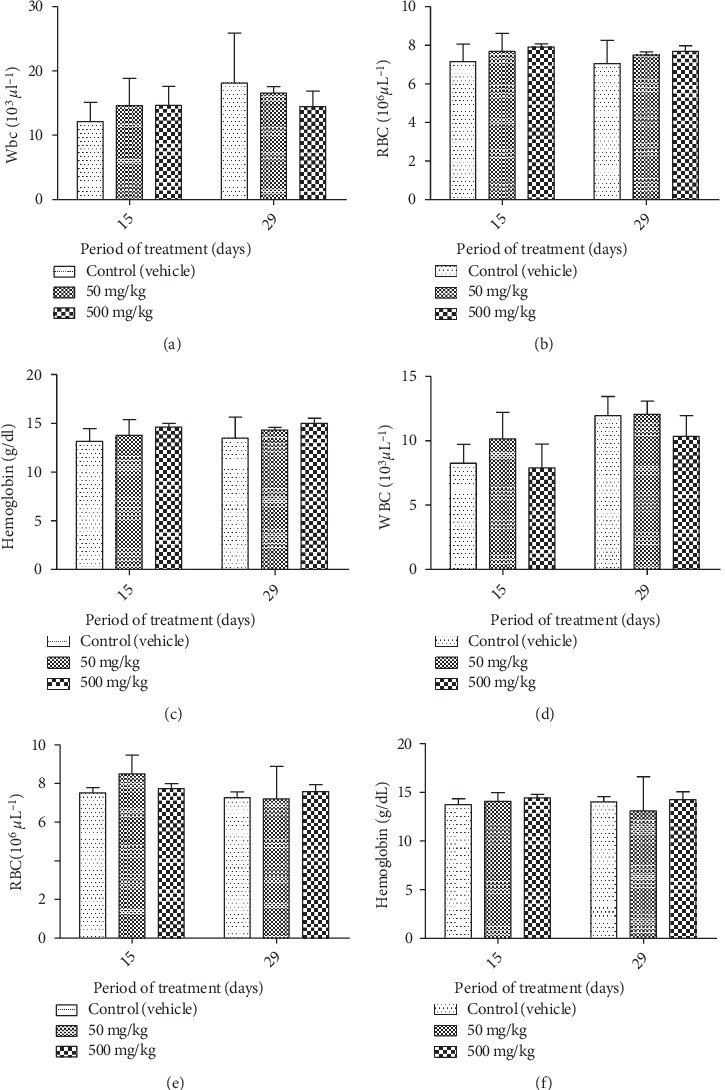
Effect of chronic oral administration of EOCG on hematological parameters in rats. The EOCG was given daily by oral route to groups of Wistar rats at the following doses: 0 (Control), 50 and 500 mg/kg for 28 days. Hematological parameters were measured at day 15 and after 28 days of treatment. (a) White blood cell (WBC) for female rats; (b) red blood cell (RBC) for female rats; (c) hemoglobin for female rats (d) white blood cell (WBC) for male rats; (e) red blood cell (RBC) for male rats; (f) hemoglobin for male rats. Data are expressed as mean ± SEM.

**Figure 8:**
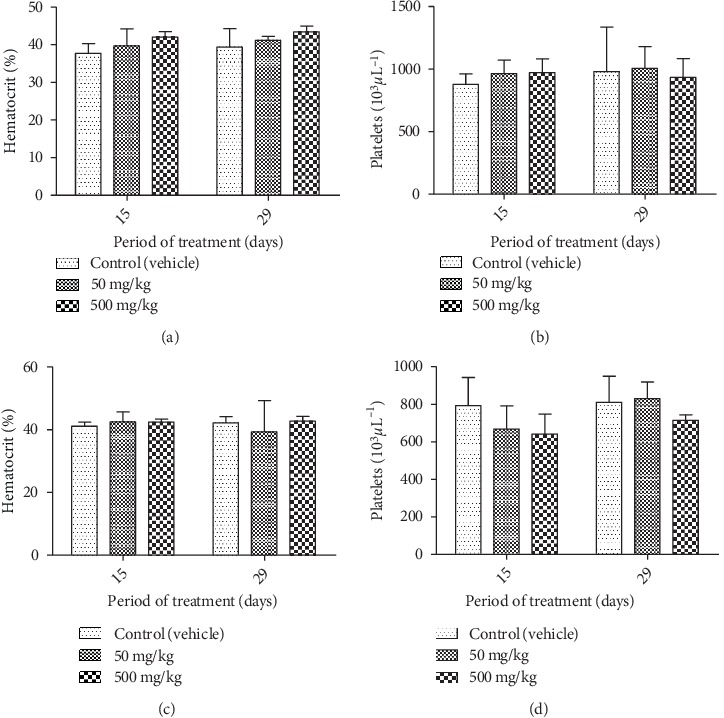
Effect of chronic oral administration of EOCG on hematological parameters in rats. The EOCG of was given daily by oral route to groups of Wistar rats at the following doses: 0 (Control), 50 and 500 mg/kg for 28 days. Hematological parameters were measured at day 15 and after 28 days of treatment. (a) Hematocrit for female rats; (b) platelets for female rats; (c) hematocrit for male rats; (d) platelets for male rats. Data are expressed as mean ± SEM.

The general behavior of rats was not affected by the EOCG administration for 28 days at 50 and 500 mg/kg. We observed no death and no significant clinical signs during the test. Administration of EOCG did not modify body weight gains of treated rats compared to controls (Figure 3). Concerning the relative weight of liver, kidney and stomach at the end of the test, there was no significant difference between control and treated groups (Figure 4). EOCG did not induce any significant variation in clinical chemistry parameters. Liver enzymes (ASAT, ALAT and PAL) and renal parameters (urea and creatinine) were not different in treated rats compared to controls (Figures 5 and 6). The hematological parameters such as hemoglobin, hematocrit, red blood cells count, platelet count, total and differential leukocytes count were similar in EOCG treated groups compared to controls (Figures 7 and 8).

**Figure 9:**
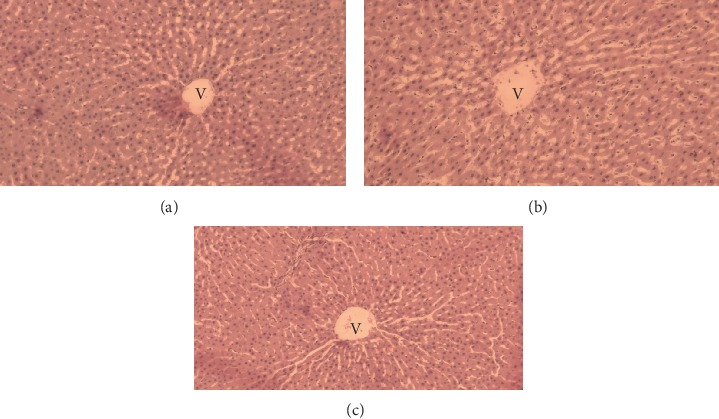
Liver of control (a) and treated rats with the EOCG at 50 mg/kg (b), and 500 mg/kg (c) body weight after hematoxylin and eosin staining (×40 magnification) showing general vision of normal hepatic lobule relating hepatocytes disposed radially around centrolobular vein (V).

**Figure 10:**
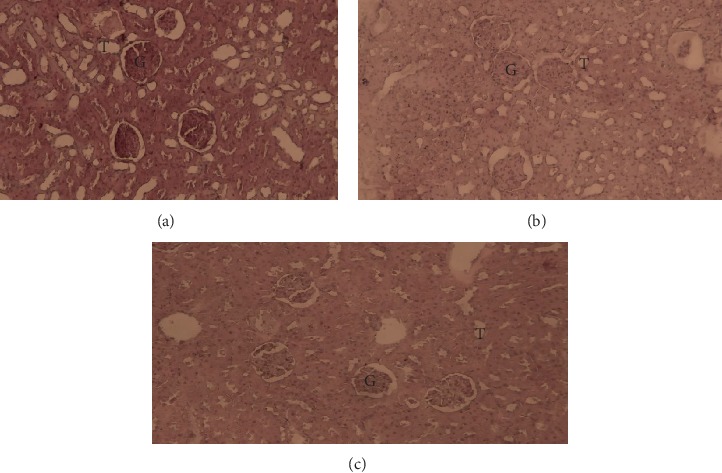
Kidney of control rats (a) after hematoxylin and eosin staining (×40 magnification) showing the renal cortex with normal glomeruli (G) and tubules (T) and treated rats with the EOCG at 50 mg/kg (b) and 500 mg/kg (c) body weight.

A histopathological examination of liver and kidneys tissues did not reveal any abnormality and/or any difference compared to the control group. These results showed that EOCG administrated orally at 50 and 500 mg/kg b.w. did not induce gross adverse effect in these organs (Figures 9 and 10).

### 3.4. Acute Pulmonary Toxicity Tests

**Figure 11:**
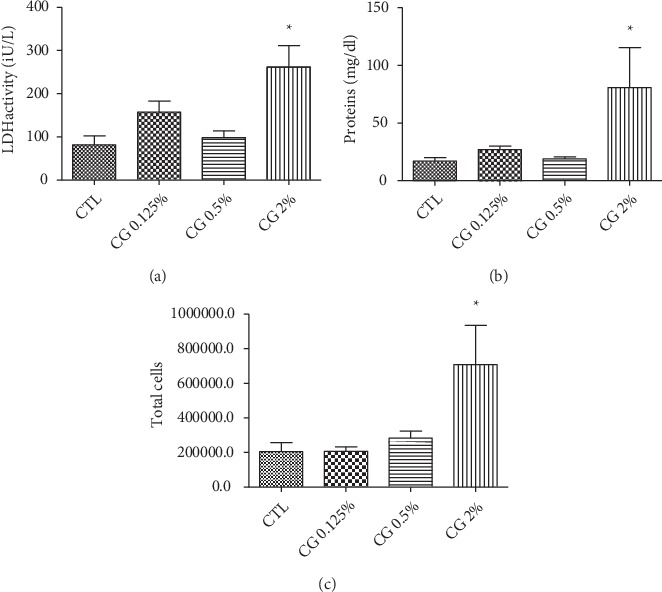
Effect of single administration of 300 *µ*l of emulsion of EOCG at 0.125 (CG 0.125%), 0.5% (CG 0.5%) or 2% v/v (CG2%) by oropharyngeal aspiration on LDH activity (a), proteins activity (b) and total cells measured (c) in BALF (Broncho Alveolar Lavage Fluid) after 3 h. Each condition was compared to control (CTL). [^∗^*p* < 0.05]. One-way ANOVA followed by a Dunnett's multiple comparison, *n* = 5, means ± SEM).

**Figure 12:**
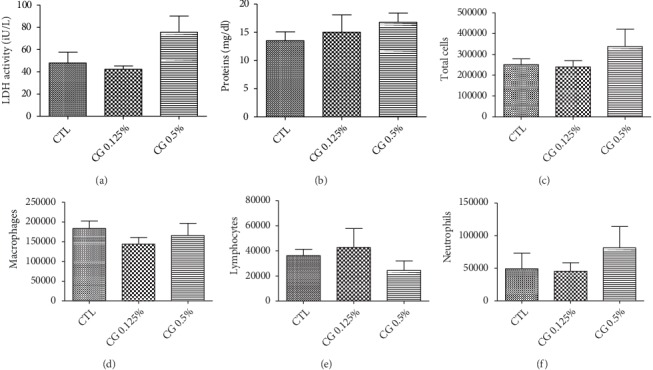
Effect of single administration of 300 *µ*l of emulsion of EOCG at 0.125% v/v (CG 0.125%) or 0.5% v/v (CG 0.5%) by oropharyngeal aspiration on total proteins (a) and LDH activity (b), total cells (c), macrophage (d), lymphocytes (e), and neutrophils (f) measured in BAL (Broncho Alveolar Lavage) after 24 h; each condition was compared to control (CTL). *p* > 0.05 for all experiments. One-way ANOVA followed by a Dunnett's multiple comparison, *n* = 5, means ± SEM).

In the first set of tests, a single administration of 300 *µ*l emulsion of EOCG at 5% v/v caused death of all rats of the group just 10 min after administration while the group of rats which received EOCG at 2% v/v showed a significant increase of total proteins, LDH activity and total cells but no death was observed 3 h after treatment. A single dose of 0.125% or 0.5% v/v did not induce any deaths 3 h after treatment (Figure 11). Indeed, these administrations provoked a slight but not significant increase of all parameters compared to control. The absence of significant toxicity at these concentrations was confirmed in the second set of tests (measurements 24 h after administration) with no significant increase of total proteins and LDH activity, total cells neutrophils, macrophages and lymphocytes (Figure 12).

## 4. Discussion

Medicinal herbs have an important place in the healthcare system of developing countries, such as in Sub-Saharan Africa. Even though toxicological evaluations are necessary to determine the safety of their use, very little has been done, so far, in this direction. Although *Cymbopogon giganteus *plant was used in traditional medicine for several beneficial properties, to the best of our knowledge, there is no data in the literature on mutagenesis or *in vivo* toxicity on EOCG.

In the present study, the *in vitro* mutagenic activity of emulsions of EOCG was assessed at five concentrations (200, 50, 20, 5, and 2 *μ*g/plate). EOCG did not induce an increase in the number of revertant colonies compared to control in any of the two strains either with or without S9 metabolic activation. The results obtained were in accordance with literature data indicating that most essential oils do not have mutagenic properties [26]. Furthermore, some authors have reported the lack of mutagenicity of limonene, which is one of the major constituents of EOCG [27].

The results of acute toxicity test did not show any mortality of animal at the dose of 2000 mg/kg, showing that the LD_50_ is above this limit. There is no data about oral toxicity of pure EOCG main components in the literature except for limonene. Indeed, limonene itself also has a median lethal dose (LD_50_) higher than 2000 mg/kg [28]. Furthermore, it has been reported that essential oils rich in carvone, e.g. spearmint (*Mentha spicata*), were characterized by a low acute toxicity (LD_50_ > 2000 mg/kg) [29]. According to the globally harmonized system (GHS) classification, the EOCG should be classified in the category 5 since the LD_50_ is estimated to be over 2000 mg/kg (corresponding to the lower toxicity category). Following repeated exposure at doses of 50 mg/kg and 500 mg/kg for 28 days, the lack of change in body weight can be interpreted as a good index of safety of the tested compound [30]. No general behavior changes were observed in treated groups compared to control group, suggesting that the chronic use of EOCG did not affect the normal growth of rats or induce any adverse effect. Atrophy of organs during this type of test can be interpreted as a toxic effect of the tested compound [31]. Since no reduction in organ weight (liver, kidneys and stomach) was detected during the 28 days for EOCG, it could be postulated that EOCG did not induce any harmful effect during the test period. However, this result is not sufficient to attest to the total safety of EOCG on these organs. The serum levels of liver enzymes and kidney biomarkers are also important parameters to assess the toxicity of a studied compound. The hepatotoxicity was assessed by determination of serum enzymes such as ASAT, ALAT and PAL. The results show that chronic ingestion of EOCG at 50 mg/kg and 500 mg/kg did not affect liver function. Urea and creatinine, known parameters of kidney function [32] were not perturbed by the daily ingestion of 50 mg/kg and 500 mg/kg of EOCG, suggesting that the use of EOCG at these doses is safe. These findings were confirmed by histopathological examinations of the organs studied, showing normal architecture of the histological structure. Measuring blood parameters is an excellent index of adverse effects on hematopoietic system which is one of the most sensitive targets for toxic compounds [33]. The oral administration of EOCG, even at 500 mg/kg daily for 28 days did not affect the hematological parameters in both genders. Considering all these data and comparing to other well-known uses and toxic EOs such as EO of *Chenopodium ambrosoïdes* (LD_50 _= 250 mg/kg) or EO of *Peumus boldus *(LD_50 _= 130 mg/kg) [34], EOCG can be considered as well tolerated by oral route at tested concentrations.

The lung is a highly vascularized organ and is also a target organ for toxicity of natural or chemical substances in general [35]. Knowledge of the toxic effects of phytomedicines on lungs, particularly those based on essential oils which are volatile, could establish its safety of use. The concentrations tested showed antibacterial and anti-inflammatory efficiencies but also corresponded to the concentrations commonly used in aromatherapy [36]. To assess the toxicity of this EO, which could reach lungs when used in sprays or fumigations, pulmonary toxicity was assessed by oropharyngeal aspiration. This technique, because of its ease and low-cost of implementation, its noninvasive nature and its ability to produce the same effects as an inhalation test, remains a good technique for the evaluation of deleterious effect on the lungs [37]. Indeed, the BALF allows to measure parameters which inform us about a possible toxicity. An increase in total proteins and LDH activity corresponds to cell toxicity and increase of alveolo-capillary permeability, respectively, of the tested substance. Likewise, an increase of the total cells with an important cellular recruitment (macrophages and neutrophils), is a sign of inflammation [37]. Administration of EOCG at concentration of 5% v/v caused death of all rats while administration of EOCG at 2% v/v increased significantly all measured parameters. There is no data in literature to compare the pulmonary toxicity of this EO with others. However, these findings suggest the lethality of EOCG at high concentrations. Administrations performed at 0.125% v/v and 0.5% v/v did not significantly increase these parameters 3 h or 24 h after administration. This is the first report on pulmonary toxicity of EOCG by oro-pharyngeal aspiration. Nevertheless, we have to notice that this technique only allows to assess the toxic effects on the lower part of the respiratory tree.

## 5. Conclusions

Essential oils have to be used with caution because of the potential toxic effects of some of them [34]. Therefore, toxicological studies should be always performed to determine the safe doses for each essential oil. The present study showed the absence of *in vitro* mutagenicity of EOCG at the maximum dose tested. Acute and subchronic toxicity assessment of EOCG did not show any mortality or deleterious effects at all tested concentrations. Finally, the acute pulmonary toxicity assessed by oropharyngeal administration did not show a significant toxicity at 0.125% v/v and 0.5% v/v but higher concentrations were toxic. Taking together, our results show that EOCG have a very low potential to generate adverse effects at doses lower than 0.5% v/v but higher doses may be toxic. However, more additional toxicological studies on other organs and functions such as reproduction are needed to complete the safety profile of this EO.

## Figures and Tables

**Table 1:** Results of AMES test for EOCG with *Salmonella typhimurium* TA98 and TA100.

Strain	TA98					
	Without S9 mix			With S9 mix		
Concentration (*µ*g/plate)	Mean	SD^d^	*N* ^a^	Mean	SD^d^	*N*
200	26	3	1.35	25.34	2.08	0.88
50	19.67	4.94	1.02	23.67	3.79	0.82
20	26	9.54	1.35	32.34	6.81	1.12
5	22.67	2.09	1.17	26	6.56	0.90
2	27.34	0.57	1.41	28	2.65	0.97
Positive control^b^	392	43.32	20.28	326.67	66.50	11.27
Vehicle control^c^	19.34	4.51	1	29	9.17	1
Spontaneous colonies	25.34	7.37	1.31	26.34	7,58	0.91

Strain	TA100					
	Without S9 mix			With S9 mix		
Concentration (*µ*g/plate)	Mean	SD^d^	*N * ^a^	Mean	SD^d^	*N*

200	146	6.10	0.99	178	10.15	0.98
50	149	13.12	1.01	172.34	4.51	0.95
20	147	19.80	0.1	185	16.65	1.02
5	147.67	11.93	1	196	4.59	1.08
2	160	5.57	1.09	185.34	21.60	1.02
Positive control^b^	1378.67	48.23	9.34	736	117.85	4.04
Vehicle control^c^	147.67	6.37	1	182.34	6.43	1
Spontaneous colonies	157.34	8.15	1.07	173.64	11.60	0.96

*Note*: ^*a*^*N*: number of colonies obtained for the test substance/number of colonies obtained for the vehicle control. ^b^Positive control: 2 *μ*g/plate of sodium azide (TA100) and 0.2 *µ*g/plate of 4-nitroquinoline-*n*-oxide (TA98) for test without S9 mix and 1 *μ*g/plate of 2-aminoanthracene for test with S9. ^c^Vehicle control: solution of Tween 80 (20 *µ*g/plate). ^d^SD: standard deviation.

## Data Availability

The data used to support the findings of this study are included within the article.
